# Machine learning applications in craniosynostosis diagnosis and treatment prediction: a systematic review

**DOI:** 10.1007/s00381-024-06409-5

**Published:** 2024-04-22

**Authors:** Angela Luo, Muhammet Enes Gurses, Neslihan Nisa Gecici, Giovanni Kozel, Victor M. Lu, Ricardo J. Komotar, Michael E. Ivan

**Affiliations:** 1 Department of Neurosurgery, Miller School of Medicine, University of Miami, 1475 NW 12th Ave, Miami, FL 33136 USA; 2 Hacettepe University School of Medicine, Ankara, Turkey; 3 Warren Alpert Medical School, Brown University, Providence, RI USA

**Keywords:** Craniosynostosis, Machine learning, Pediatric neurosurgery, Diagnostic models, Treatment outcome prediction

## Abstract

Craniosynostosis refers to the premature fusion of one or more of the fibrous cranial sutures connecting the bones of the skull. Machine learning (ML) is an emerging technology and its application to craniosynostosis detection and management is underexplored. This systematic review aims to evaluate the application of ML techniques in the diagnosis, severity assessment, and predictive modeling of craniosynostosis. A comprehensive search was conducted on the PubMed and Google Scholar databases using predefined keywords related to craniosynostosis and ML. Inclusion criteria encompassed peer-reviewed studies in English that investigated ML algorithms in craniosynostosis diagnosis, severity assessment, or treatment outcome prediction. Three independent reviewers screened the search results, performed full-text assessments, and extracted data from selected studies using a standardized form. Thirteen studies met the inclusion criteria and were included in the review. Of the thirteen papers examined on the application of ML to the identification and treatment of craniosynostosis, two papers were dedicated to sagittal craniosynostosis, five papers utilized several different types of craniosynostosis in the training and testing of their ML models, and six papers were dedicated to metopic craniosynostosis. ML models demonstrated high accuracy in identifying different types of craniosynostosis and objectively quantifying severity using innovative metrics such as metopic severity score and cranial morphology deviation. The findings highlight the significant strides made in utilizing ML techniques for craniosynostosis diagnosis, severity assessment, and predictive modeling. Predictive modeling of treatment outcomes following surgical interventions showed promising results, aiding in personalized treatment strategies. Despite methodological diversities among studies, the collective evidence underscores ML’s transformative potential in revolutionizing craniosynostosis management.

## Introduction

Craniosynostosis is a complex diagnosis that refers to the premature fusion of one or more of the fibrous joints which connect the bones of the skull, also known as the cranial sutures [1, 2]. The condition can be subdivided according to the specific sutures which prematurely fuse and includes the classifications of sagittal, seen in approximately 60% of patients, coronal in 25% of patients, metopic in 15% of patients, and lambdoid, seen in 2% of patients [3]. In certain cases, craniosynostosis can result in serious complications, including developmental delay, facial abnormality, sensory, respiratory, and neurological dysfunction, anomalies affecting the eye, and psychological disturbances [4]. Due to these potentially life-altering outcomes, the early identification and diagnosis of craniosynostosis is vital for successful treatment.

Because the recognition of craniosynostosis before symptoms emerge relies on a physician’s abilities to determine whether or not the physical dimensions of a patient’s skull falls outside a normal range of values, there is great hope that this kind of diagnosis can be automated and improved with the help of machine learning (ML) [5, 6], an area of research which is currently showing great merit in the field of neurosurgery and neuro-oncology [7–10]. Each type of synostosis has characteristic skull measurements that ML programs can be trained to recognize by reading the imaging data for a patient and finding the necessary measurements of the skull needed to determine whether or not the patient is likely to have a type of synostosis [7]. The end goal of this training is to develop a reliable ML model which possesses an accuracy and speed of diagnosis of synostosis which is greater than that of human physicians.

This systematic review discusses the kinds of ML models and methodologies currently being investigated for clinical use in the diagnosis of craniosynostosis and also examines the advancements of 3D imaging technology in conjunction with artificial intelligence for greater identification of pathologies. Thirteen papers were interrogated to this effect, and the collective results of its authors’ work has been assembled for a clear and concise overview of the state of research on this topic.

## Methods

A comprehensive search was conducted on the PubMed and Google Scholar databases to identify relevant studies focusing on the application of ML in craniosynostosis. The search strategy employed a combination of keywords. The search string utilized terms related to “craniosynostosis,” “Machine Learning,” “3D Imaging,” and relevant synonyms. Boolean operators (AND, OR) were used to refine the search.

### Inclusion and exclusion criteria

Studies were considered eligible for inclusion if they met the following criteria: (1) Published in peer-reviewed journals, (2) written in English, (3) investigated the utilization of ML algorithms, models, or techniques in the context of craniosynostosis diagnosis, prognosis, treatment, or outcome prediction, and (4) included human subjects or clinical data related to craniosynostosis Exclusion criteria encompassed studies that were conference abstracts, letters, or duplicates, as well as those not focused on craniosynostosis or lacking ML applications.

### Study selection

The initial search results were screened based on titles and abstracts to identify potentially relevant studies. Full-text assessment was performed for articles meeting the inclusion criteria. Three independent reviewers conducted the screening and selection process, with any discrepancies resolved through discussion or consultation with a fourth reviewer if needed.

### Data extraction

Two independent reviewers performed data extraction from the selected studies using a standardized data extraction form specifically developed for this systematic review. The extraction form was designed to capture key elements relevant to the utilization of ML in craniosynostosis research. The key elements include study characteristics (authors, publication year), study design, ML algorithms employed, dataset descriptions, outcome measures, and key findings related to craniosynostosis. Data extraction was cross-checked for accuracy and completeness by both reviewers, with any discrepancies resolved through discussion and consensus.

### Bias assessment

Risk of bias assessment was performed using the Joanna Briggs Institute (JBI) checklists for case series and randomized controlled trials [11].

## Results

**Fig. 1:**
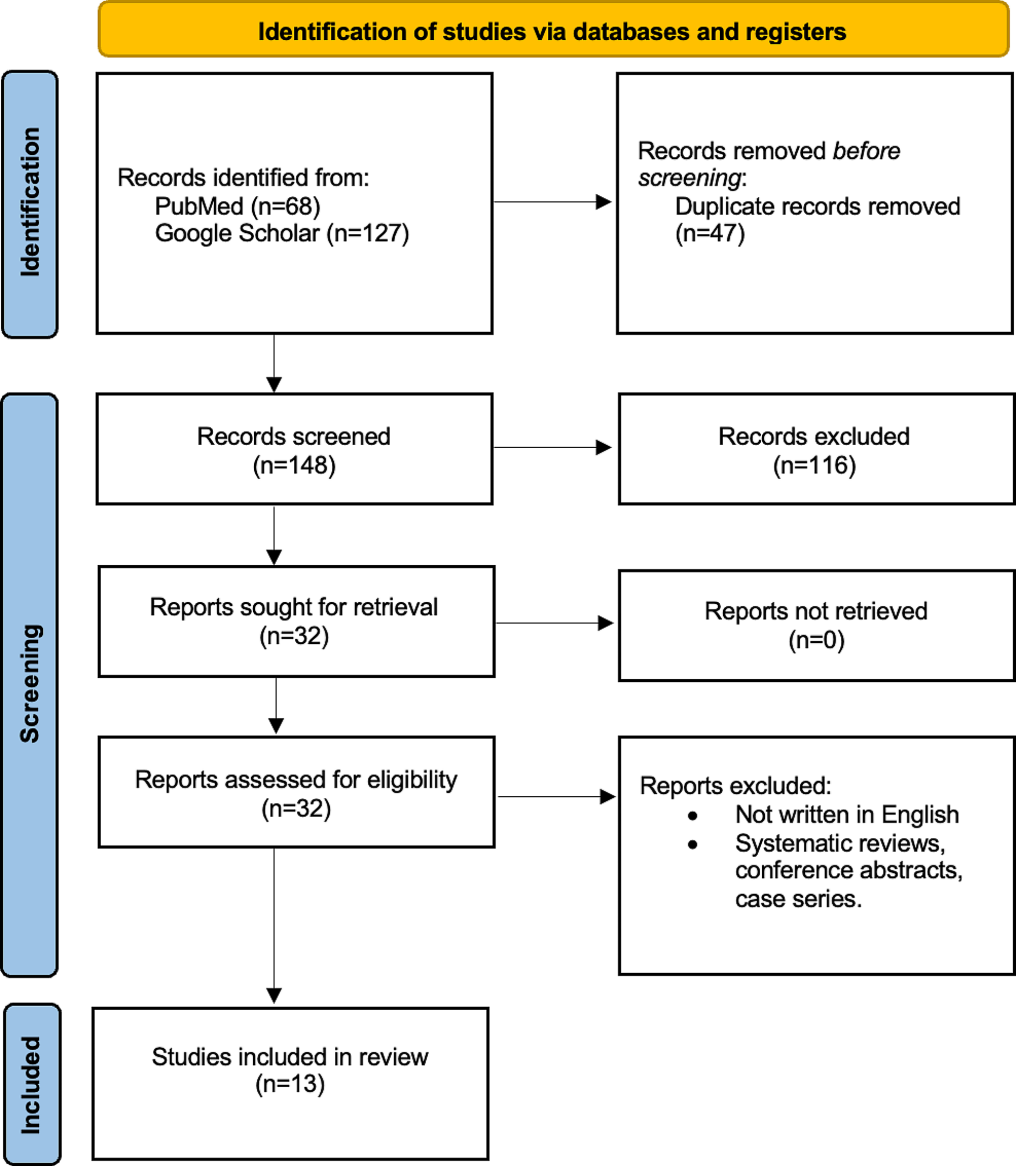
PRISMA flow diagram

**Fig. 2:**
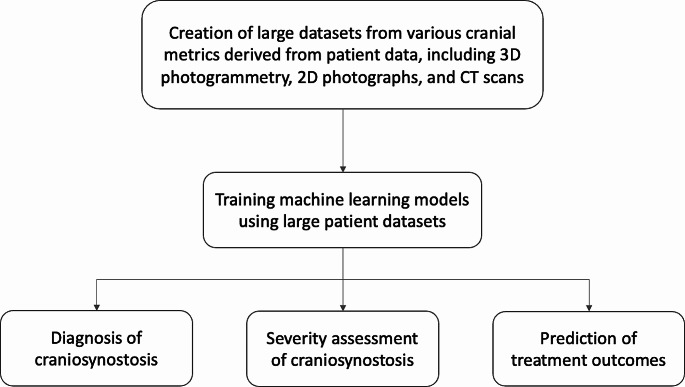
Flow diagram depicting the method of extracting data, training models, and its use in craniosynostosis

PRISMA flow diagram of the literature search and study selection was demonstrated in Fig. 1. Literature search yielded 148 citations after removing duplications. 13 of these citations were identified as eligible and included in the study according to inclusion criteria (Table 1) [12–24]. Figure 2 summarizes the applications of ML in the management of craniosynostosis.The JBI criteria-based assessment for risk of bias revealed that all the studies included had a low risk of bias (Supplementary File 1).

**Table 1:** Summary of the Studies Utilizing ML Techniques for Craniosynostosis Diagnosis and Classification

Study	Number of patients	Age of patients	Surgical Technique Used	Metric Used	Algorithm
Anderson et al. 2023 [12]	1001 (sagittal craniosynostosis *n* = 122, other cranial deformity *n* = 565, normocephalic = 314).	171.2 ± 72.7 days.	NR	Posterior Arc Angle (PAA) [compared to Cephalic Index (CI)]	Tree Based ML Model
Anstadt et al. 2023 [13]	124 (30 with MCS, 94 controls)	5–15 months	NR	Cranial Morphology Deviation (CMD)	Unsupervised ML algorithm implemented through ShapeWorks software
Bhalodia et al. 2020 [14]	17 affected, 65 non-affected	5–15 months	NR	Shape Descriptor [compared to Interfrontal Angle (IFA)]	Maximum Likelihood Estimation ML
Blum et al. 2023 [15]	39	7.7 ± 3.4 months	Bifrontal orbital advancement and remodeling	Metopic Severity Score (MSS) and Cranial Morphology Deviation (CMD)	CranioRate: a dysmorphology quantification tool based on unsupervised ML model
Bookland et al. 2021 [16]	40	136.7 ± 78.2 days	NR	Cephalic Index (CI), Cranial Vault Asymmetry Index (CVAI), PAA, sagittal Hu	Linear Discriminant Analysis
Bruce et al. 2022 [17]	16 patients, 11 controls	0.97 ± 0.28 years	NR	MCS Severity Scores	Principal Component Analysis Principal Component Regression
Cho et al. 2018 [18]	43 (16 underwent surgical treatment, 27 conservative treatment)	Surgical treatment group: 5.9 months Non-surgical Treatment group: 8.3 months	NR	Average mean curvature of: the mid-forehead strip and the right/left supraorbital areas	K means clustering
Junn et al. 2021 [19]	194 CT scans analyzed (167 metopic CS and 27 controls)	Metopic CS: 7.18 ± 4.70 months Controls: 9.21 ± 7.82 months	NR	Metopic Severity Score	Unsupervised ML algorithm
Paro et al. 2022 [20]	174	160 days	NR	CI, CVAI, Anterior Arc Angle (AAA), Transcanthal Line Angle (TCLA)	Random Forest Partition Tree model Classification and Regression tree Linear Discriminant Analysis
Porras et al. 2019 [21]	266 controls, 201 with Craniosynostosis	Control: 1.93 ± 1.69 years Craniosynostosis patients: 0.77 ± 1.29 years	NR	Malformations and Curvature Discrepancies	Support Vector Machine Classifier
Sabeti et al. 2022 [22]	145	Under 1 year old	Minimally invasive suturectomy or open cranial vault reconstruction	CI, CVAI, Anterior-Midline Width Ratio (AMWR), Anterior-Posterior Width Ratio (APWR), Left-Right Height Ratio (LRHR)	Linear Discriminant Analysis k-Nearest Neighbor Support Vector Machine Random Forest Bagging (Ensemble method)
Schaufelberger et al. 2022 [23]	367	Under 1.5 years old	NR	Data from 3D photogrammetry scans	Support Vector Machine Linear Discriminant Analysis Naive Bayes Bagged Decision Trees k-Nearest Neighbors
Villavisanis et al. 2022 [24]	124	3.59 ± 0.87 months	Spring-mediated cranioplasty	CI, Spring Length and Width, and Parietal Bone Thickness	Stepwise Multiple Regression Analysis Least Absolute Shrinkage and Selection Operator Random Forest

### Detection of craniosynostosis

Several papers focused on the detection of craniosynostosis using ML models. Anderson et al. introduced a model combining parietal angle (PAA) with cranial index (CI), increasing sensitivity in identifying sagittal craniosynostosis. In 6 of 122 cases, PAA was abnormal while CI was normal, highlighting the potential of adding PAA to improve detection. The model achieved an overall accuracy of 89.3% [12]. Bookland et al. developed a telehealth-compatible diagnostic software with 93.3% accuracy, 92.0% sensitivity, and 94.3% specificity [15]. Paro et al. demonstrated the accuracy of pretrained ML models in identifying craniosynostosis in outpatient clinics, with the best-performing model achieving 94.8% accuracy, 87.0% sensitivity, and 96.0% specificity. Studies also emphasized radiation-free approaches for diagnosis and classification [20]. Schaufelberger et al. presented a shape-model-based classification pipeline with an accuracy of 97.8% in diagnosing craniosynostosis. Their statistical shape model performed similarly to those based on CT scans and stereophotogrammetry [23].

### Severity assessment of metopic craniosynostosis

Several studies delved into quantifying the severity of and evaluating metopic craniosynostosis. Anstadt et al. and Bhalodia et al. utilized unsupervised ML and shape analysis to objectively measure severity, showing correlations with clinical assessments [14]. Anstadt’s study included 36 craniofacial surgeons and found better correlation with 3D shape analysis compared to interfrontal angle assessments [13]. Blum et al. explored the impact of preoperative phenotypic severity on long-term aesthetic outcomes, revealing a significant negative correlation between severity scores and age at computed tomography [15]. Bruce et al. presented 3D photography as a valid alternative to CT scans for evaluating metopic craniosynostosis. Their study, including 14 patients, demonstrated a close correlation between ML algorithm predictions from 3D photographs and CT scans [17].

### ML for identification and diagnosis of craniosynostosis

ML played a crucial role in identifying and diagnosing craniosynostosis across various studies. Cho et al. used an unsupervised ML algorithm to differentiate between benign metopic ridge and true metopic craniosynostosis, achieving 96% agreement with surgeons’ decisions [18]. Sabeti et al. developed a user-friendly diagnostic system for newborns using digital photos, achieving classification results between 85% and 92%, statistically higher than handcrafted indices [22]. Junn et al. assessed the diagnostic concordance between a ML-derived algorithm and manually measured severity indices, demonstrating a high diagnostic value of the ML-derived score comparable to other severity indices [19].

### Predictive model for Surgical outcomes

Blum et al. studied a total of 39 patients who underwent bony fronto-orbital advancement reconstruction (BFOAR) at an average age of 9.9 months between 2012 and 2017. The average Metopic Severity Score (MSS) of these patients was 6.3 out of 10, indicating an average severity relative to the larger metopic population. Regarding surgical technique, most patients (84.6%) received interpositional bone grafts to the central bandeau, with 31.4% receiving parietal bone grafts. The average width of the interpositional bone graft was 17 mm, with wider grafts typically seen in more phenotypically severe patients. Esthetic assessment conducted at an average of 5.4 years post-operation revealed the presence of vertical indentation (VI) in 87.2% of patients, temporal hollowing (TH) in 76.9%, frontal bone irregularity (FBI) in 61.5%, and lateral orbital retrusion (LOR) in 20.5%. The majority of patients (61.5%) received a median Whitaker classification of II, indicating some degree of esthetic irregularity. Secondary operative intervention was required in only 5.2% of patients, primarily for relapse, with two additional patients considering revision surgery in the future. The Metopic Severity Score (MSS) was identified as the only independent predictor of vertical indentation (VI), while younger age at surgery and increased length of follow-up were associated with worse Whitaker scores, indicating poorer esthetic outcomes. Operating at a later age may potentially lead to better esthetic outcomes, although this decision is complex and requires consideration of various factors. Additionally, increased length of follow-up was linked to worse esthetic outcomes, highlighting the importance of continued longitudinal monitoring to assess the quality of correction and identify any late-onset esthetic issues [15].

Villavisanis et al. explored predictive modeling for surgical outcomes following spring-mediated cranioplasty (SMC). The study included 124 patients with nonsyndromic sagittal craniosynostosis undergoing SMC between 2014 and 2021. SMC involved the placement of cranial torsional springs along the sagittal suture to facilitate cranial expansion. The springs were positioned anteriorly, posteriorly, and sometimes in the middle of the parietal bones to promote skull reshaping. The majority of patients received three springs, with varying forces and lengths. The springs remained in place for an average of 3.7 months postoperatively. The study reported relatively low estimated blood loss during surgery and a low rate of intraoperative transfusion. The average duration of the SMC procedure was around 104 min, and the average length of hospital stay was less than 2 days. The cephalic index (CI) increased significantly postoperatively, indicating successful cranial reshaping. The changes in CI were most pronounced in the immediate postoperative period and tended to plateau over time, with long-term stability observed up to 5 years postoperatively. Analysis of preoperative imaging revealed variations in parietal bone thickness among patients. While parietal bone thickness was implicated in some predictive models, it was not a primary factor driving changes in CI. Maximum and total spring forces, anterior and posterior spring lengths, and duration were identified as the most predictive variables for changes in cephalic index, with demographic variables being inferior predictors [24].

## Discussion

Sagittal and metopic craniosynostosis are the first and third most common types of craniosynostosis in infants [25–27]. However, the reason that metopic rather than sagittal craniosynostosis appears to have a greater area of research in ML identification is due to the fact that metopic craniosynostosis is slightly harder to identify visually than sagittal craniosynostosis. Sagittal craniosynostosis results in scaphocephaly, in which the skull is elongated and narrowed along the sagittal suture, while metopic craniosynostosis results in trigonocephaly, which presents as a triangular-shaped head that is narrow at the front of the skull and broader at the back [28, 29]. Metopic craniosynostosis therefore imitates a natural head shape more closely than sagittal craniosynostosis, and, as a result, is more difficult to detect on visual inspection. This makes the utilization of a computer program which can quickly and objectively measure skull shape parameters and compare them to values defined as normal quite useful in metopic craniosynostosis identification rather than sagittal craniosynostosis.

The two sagittal craniosynostosis papers examined in this study utilized the head shape parameter of CI as the variable of interest for their ML models. CI is an incredibly useful metric in the evaluation of neonatal skull shapes, particularly when planning potential surgical procedures for children with cranial deformations [26]. It is defined as the width of the skull divided by the length of the skull, multiplied by one hundred. Villavisanis et al. created an algorithm to analyze the changes in CI which occur after spring-mediated cranioplasty, one of the four main surgical interventions for the treatment of craniosynostosis. Spring placement, anterior spring force, and anterior spring length were all found to be statistically significant predictors of changes in CI upon multivariate analysis [24]. Anderson et al. were attempting to improve CI as a variable for the prediction of sagittal craniosynostosis by adding a new variable which they called the PAA, which represents biparietal narrowing of the skull, to a tree-based ML model that predicted sagittal craniosynostosis. This model was demonstrated to have an increased accuracy when operating with both CI and PAA as variables than with CI alone, indicating that CI, while already a reliable predictor of sagittal craniosynostosis, can be improved with additional parameters in a ML model [12].

For the six papers that examined metopic craniosynostosis, three utilized the MSS for their ML models, two utilized CMD, and three utilized novel variables designed or chosen specifically by those studies. The MSS is actually a composite score assigned by a ML algorithm which represents the severity of the patient’s head shape based on characteristic features of metopic synostosis. All three studies which used MSS also all utilized the ShapeWorks software suite to construct their ML algorithms for the calculation of their MSS scores. CMD is another composite score which can be produced by ML programs built in the ShapeWorks software suite, and its function is very similar to MSS. The main difference between these two metrics is the exact feature of the skull that they are examining and the way their scoring systems are to be read. MSS evaluates how similar a given skull is to a standard metopic skull, with a score of zero to ten being reported in each case, zero indicating a normal skull. CMD measures skull shape abnormality broadly, across several different measurements, and as a result, exists on a much larger 300 point scale, with 85 representing a normal skull. Both of these metrics were demonstrated to be effective predictors of metopic craniosynostosis across the three studies which used MSS and CMD in this fashion [13, 17, 19].

This study also found that there was a high agreement among surgeons in their calculated severity rankings for the seventeen metopic skulls examined. Cho et al. were concerned with the question of surgeons’ threshold for operative intervention in metopic craniosynostosis at one tertiary care craniofacial center. They used a novel curvature analysis in order to automatically classify cranial deformities as being either a benign metopic ridge or a true metopic craniosynostosis to evaluate the 43 patients evaluated by five surgeons over a five-year period. The surgeons studied were determined to possess a similar threshold for managing patients conservatively or surgically [18]. Thus, while no broader studies currently exist on the consistency of diagnosis and treatment of metopic craniosynostosis by neurosurgeons, these two small sample sizes indicate that there may be greater consistency among physicians than would be expected for such a difficult clinical question.

The final five papers analyzed in this review all deal with the identification of many different types of craniosynostosis by ML programs. As a result, these programs all rely on a variety of diagnostic data, including scores such as MSS and CMD previously discussed. Each of these papers also demonstrated a high accuracy for ML’s diagnosis of each type of craniosynostosis included in the studies. However, the more interesting feature of these papers is that they deal with potential further advancements in the solidifying field of ML diagnostics. Bookland et al. discussed the application of this technology of utilizing past imaging data for patients in order to make future diagnoses that did not require further imaging studies, while Porras et al. and Schaufelberger et al. examined the novel approach of using three-dimensional photography rather than traditional CT scans [16]. The former is cheap, noninvasive, and requires no exposure to x-ray radiation, all of which are advantages over the latter. The only disadvantage is that three-dimensional photography does not provide a visualization of the cranial bones and sutures in the skull [21]. However, the studies cited here have demonstrated that ML programs fed with data from three-dimensional photography still possess a very high accuracy of diagnosis, with Schaufelberger et al. finding that this accuracy was comparable to that of methods using CT scans [23]. There is therefore hope that even more safe and effective pathways of automated craniosynostosis diagnosis may be developed in the future.

### Applications to treatment

Currently, the majority of research aimed at machine learning’s application to craniosynostosis targets the diagnosis of this condition. None of the papers cited in this study attempted to apply artificial intelligence to the question of whether or not surgery is appropriate for the patient, and only a small selection of the available literature deals with the very large issue of surgical outcomes for procedures intended to rectify craniosynostosis. The two papers found by this study to examine treatment outcomes as a variable of interest were Blum et al. and Villavisanis et al. (Sabeti et al. recorded the type of treatment received by their patients but did not analyze this data in relation to skull measurements or other potentially predictive variables) [22]. Blum et al. utilized an average Whitaker classification score, calculated from the individual scores provided three craniofacial attendings, as their outcome of interest in determining whether or not treatment was successful. The Whitaker classification score is a system for describing the aesthetic outcome of craniofacial surgeries; however, it has been demonstrated that this scoring system suffers from low interrater reliability and is not predictive of future treatment [30]. As a result, the measurement of a patient’s skull aesthetic by a physician is not always the best variable to determine whether or not surgery is necessary or successful [15].

Villavisanis et al. offer an alternative approach to the assessment of craniosynostosis treatment. Their paper used the measurable Cephalic Index as the outcome of interest rather than an aesthetic score, and their work was able to determine that spring placement, anterior spring force, and anterior spring length were all predictive of changes in Cephalic Index scores for patients. These results are a good indication that machine learning models can be used to accurately determine which impact variables of interest will have on the outcome of different potential surgical interventions for patients with craniosynostosis [24]. There is also hope that, in the future, models can be designed which will predict the changes in measurable skull parameters for patients based on their current physical dimensions and the surgical options available to them. Physicians would then be able to determine which surgical option would provide the best possible outcome for the patient on the basis of changes in those parameters. However, these are currently entirely theoretical models. No such machine learning system has been publicized or shared with the scientific community with the capability of predicting surgery outcomes or assessing the probability of a particular surgical treatment’s benefit for the patient.

### Future directions and potential applications of ML in Pediatric Neurosurgery

The use of photogrammetry for 3D modeling of normal anatomy and disease-specific 3D modeling, as well as the application of artificial intelligence for the diagnosis and management of various neurological pathologies, has been widely examined in previous literature [31–34]. Additionally, several studies examined the ML’s potential as a means of pre-referral identification of and widespread routine screening for conditions such as craniosynostosis. Bookland et al. investigated a novel telehealth-compatible diagnostic software system, with the potential to be used on either a phone or computer and found that the program possessed a 93.3% accuracy in identifying craniosynostosis, with a sensitivity of 92% and specificity of 94.3%, for 339 orthogonal top-down cranial images with or without additional facial views [35]. These results not only suggest that remote screening can indeed be a substitute for optical scanner- or CT-based craniometrics, but that software can be developed for a mobile platform which will allow for screening by telemedicine or in a primary care setting, novel forms of delivery which would increase the efficacy and practicality of ML’s use in neurosurgery, allowing both families and all levels of physicians will be able to use this technology.

In addition to its application in craniosynostosis diagnosis and management, ML techniques hold promise for other areas of pediatric neurosurgery, including head circumference measurement and monitoring. Similar to the use of ML algorithms for craniosynostosis, these techniques can be trained on large datasets of head circumference measurements to develop models capable of accurately predicting normal growth patterns and identifying deviations indicative of underlying conditions such as microcephaly or macrocephaly [36]. By integrating ML-based head circumference monitoring systems into routine clinical practice, healthcare providers can potentially improve the early detection of neurological abnormalities and facilitate timely interventions to optimize patient outcomes.

Furthermore, ML models have demonstrated utility in the management of hydrocephalus, another common pediatric neurosurgical condition. A recent systematic review of ML models in normal pressure hydrocephalus (NPH) highlighted the diversity of approaches utilized, including convolutional neural networks trained on various inputs such as clinical features, imaging data (CT and MRI), and intracranial pulse waveform characteristics. These models exhibited high accuracy in diagnosing NPH and predicting patient outcomes, underscoring the potential of ML to enhance decision-making and care in hydrocephalus management [37]. However, challenges such as standardization of ML models and adherence to reporting guidelines remain, emphasizing the need for continued research and refinement in this field. Incorporating ML-based predictive models for hydrocephalus and other neurological conditions into clinical practice could aid clinicians in optimizing treatment strategies and improving patient outcomes.

### Limitations

The papers reviewed in this study represent a diverse range of methodologies in attempting to apply ML to the diagnosis and identification of craniosynostosis. As a result, this study functions best as a narrative review, rather than a meta-analysis, which cannot be performed due to the nature of the cited literature. Furthermore, retrospective data is difficult to fairly compare without more standardized prospective data in the future. As data grows regarding the application of ML to craniosynostosis diagnosis and treatment, more specific topics will be able to be evaluated.

## Conclusion

This systematic review highlights the significant strides made in utilizing ML techniques for the detection, severity assessment, and predictive modeling of craniosynostosis. Key findings underscore the promising accuracy of ML models in diagnosing craniosynostosis types, the objective quantification of severity using innovative metrics like metopic severity score and cranial morphology deviation, and the predictive modeling of treatment outcomes following surgical interventions. Despite methodological diversities among studies, the collective evidence underscores ML’s transformative potential in revolutionizing craniosynostosis management, offering efficient, radiation-free diagnostic approaches, personalized treatment strategies, and avenues for further advancements in automated diagnosis with technologies like 3D imaging. Continued research and collaboration are crucial for realizing the full clinical impact of ML in improving outcomes for individuals affected by craniosynostosis.

## Data Availability

No datasets were generated or analysed during the current study.
